# Trace metals, organic carbon and nutrients in the Beidagang Wetland Nature Reserve, northern China

**DOI:** 10.1371/journal.pone.0204812

**Published:** 2018-10-02

**Authors:** Yueqin Chen, Qiuyang Song, Ling Pan, Meiqing Jia, Congwei Li, Beibei Hu, Guanghong Wu

**Affiliations:** 1 Tianjin Key Laboratory of Water Resources and Environment, Tianjin Normal University, Tianjin, China; 2 College of Urban and Environmental Sciences, Tianjin Normal University, Tianjin, China; National Taiwan Ocean University, TAIWAN

## Abstract

This study aimed to determine sediment contamination in the Beidagang Wetland Nature Reserve to describe atmospheric deposition of trace metals. We analyzed Hg, Cd, Pb, TOC, TN, TP, δ^13^C, and δ^15^N, and studied their variations in surface sediments and in the vertical profiles of sediment cores collected from the reserve. Evaluation of environmental trace metal contamination using sediment quality guidelines and geochemical background values indicated that the risk of metal pollution in the reserve sediments was relatively low. Concentrations of Hg, Cd, and Pb in the sediments were much lower than concentrations in sediment samples from Bohai Bay and polluted rivers in Tianjin. Enrichment factors indicate that samples are moderately contaminated with Hg, Cd, and Pb; whereas the geo-accumulation index results classify the sediments as uncontaminated to moderately contaminated with Hg, Cd, and Pb. The distribution patterns of trace metal concentrations in the three core samples were uniform. δ^13^C and δ^15^N were used to track the sources of TOC and TN in sediments. Results show that TOC mainly originated from the residue and decaying matter of aquatic plants (e.g., algae, reeds, and Typha), while TN was derived from soil N and elevated atmospheric N deposition. Because domestic and industrial waste is not discharged into the Beidagang Wetland Nature Reserve, trace metals found in sediments mainly originate from atmospheric deposition. The results provide baseline data for analysis of trace metal accumulation in Beijing-Tianjin-Hebei, a region subject to atmospheric deposition in northern China.

## Introduction

Beijing-Tianjin-Hebei is the largest urbanized region in northern China, comprising the economic region surrounding the cities of Beijing, Tianjin, and Hebei, along the coast of the Bohai Sea. In this region, increasing energy production, domestic heating, and automobile traffic, have resulted in the release of considerable volumes of trace metals into the atmosphere. Thus, air quality is a major environmental issue affecting sustainable urban development and the health of inhabitants in this region. Soils in China have been contaminated with heavy metals to varying degrees. Cd and Hg have been identified as priorities for control due to their higher concentrations in soils and higher public health risks [1]. Several trace metals (e.g., Hg, Cd, and Pb) are known to be atmospheric pollutants, highly toxic to humans and wildlife even at extremely low levels. Trace metals are transported in the atmosphere either as gases or by adsorption onto particulate matter [2] and are widely distributed in the natural environment. Trace metal emissions into the atmosphere, and subsequent dry/wet deposition either directly to soils or to the surface of water bodies are important potential sources of surface water contamination in Beijing-Tianjin-Hebei. Over the last 10 years, several studies have investigated the sources and impacts of trace metal contamination in the atmosphere [3–6] and soils [7,8] of this region. However, investigations of trace metal concentrations in wetland nature reserve sediments from northern China have been limited. Wetland nature reserves are important sources of data because they are protected from human activities once they are designated as reserves, and thus sewage and wastewater cannot be discharged within their boundaries. Sediment samples from wetland nature reserves are therefore important for assessing the levels and potential sources of trace metals that have resulted from atmospheric deposition in the Beijing-Tianjin-Hebei region.

The objectives of this study were to: (1) Evaluate trace metal pollution and accumulation in the Beidagang Wetland Nature Reserve sediments; (2) Identify potential sources, and spatial and temporal variations in total organic carbon (TOC), total nitrogen (TN), total phosphorus (TP), carbon and nitrogen stable isotopes (δ^13^C andδ^15^N), Hg, Cd, and Pb inputs to the reserve, and; (3) Assess the influence of atmospheric deposition of trace metals within the reserve.

## Materials and methods

### Ethics statement

No specific permissions were required for the present study as we only collected a limited number of water and sediment samples from the nature reserve. Our field study did not involve any endangered or protected species.

### Study area

The Beidagang Wetland Nature Reserve (38°36′–38°57′ N and 117°11′–117°37′ E) is situated on the coast of Bohai Bay, southeast of Tianjin, northern China. The reserve has an area of about 34,887.13 ha and falls on a migration route vital for the annual movement of birds between the north and south of East Asia. It is an important wintering and staging area for more than 100,000 birds. The reserve is divided into three functional zones: the core region, buffer zone, and experimental zone. The core region of the reserve was founded in 1974 and has an area of about 16,400 ha [9]. Although considerable ecological changes have occurred in and around the reserve in recent years due to increased salinization and water resource shortages [10], assessment of anthropogenic impacts on the environment through quantification of trace metals (i.e., Hg, Cd, and Pb), organic carbon, and nutrients in the Beidagang Wetland Nature Reserve sediments remains very limited.

### Samples

**Fig 1:**
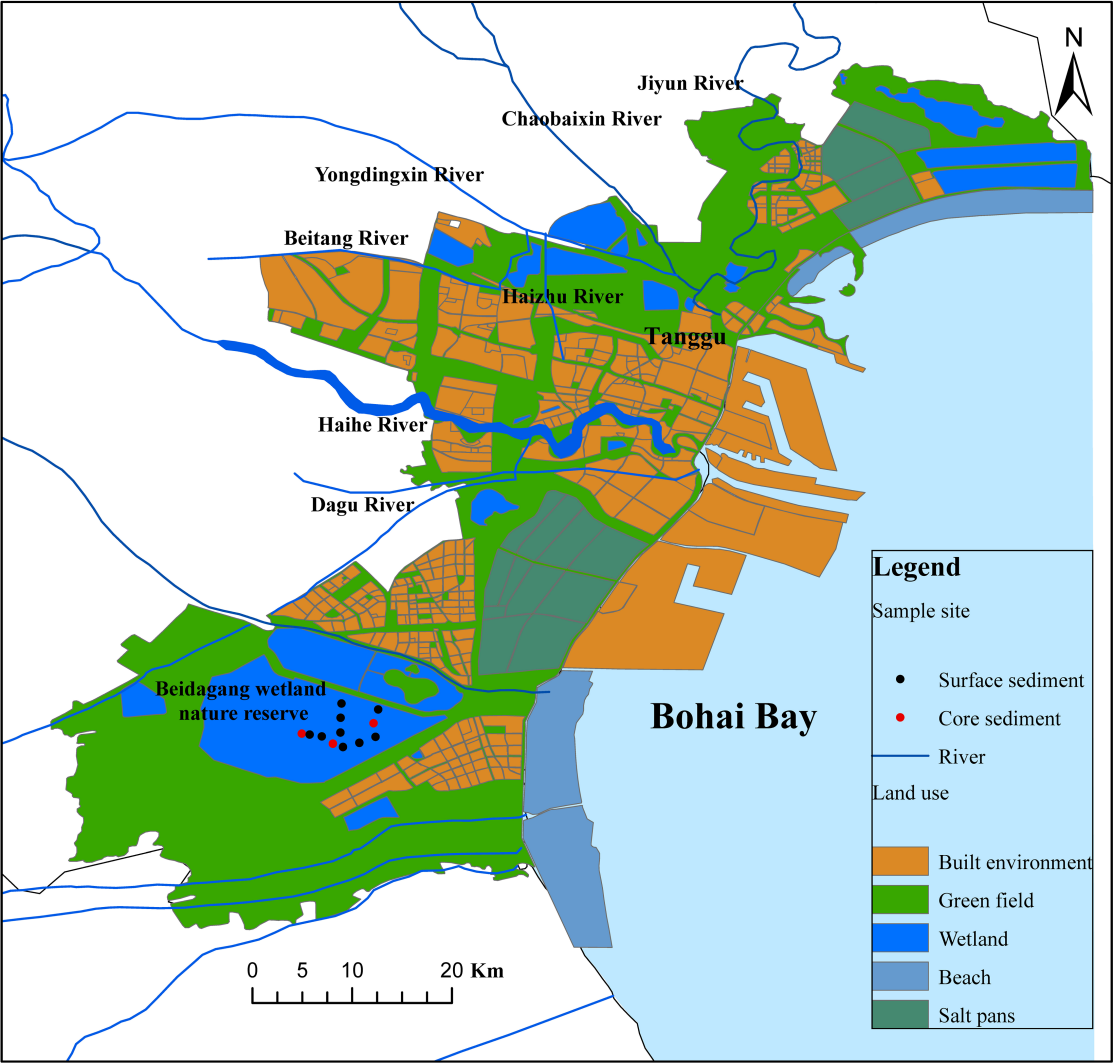
Location of sampling sites within the Beidagang Wetland Nature Reserve in northern China.

Surface sediment samples were collected in September 2014 from the top 0–2.5 cm at 12 sites and sediment profile samples were collected from the top 0–35 cm at three sampling sites, all located within the core zone of the Beidagang Wetland Nature Reserve (Fig 1). Sediment cores were then sliced at 2.5 cm intervals and immediately frozen. All samples were air-dried at 50°C overnight, passed through a 2mm stainless steel sieve to remove large debris, and then homogenized by quartering with a riffler. The resulting subsamples were ground and sieved so that they could pass through a 100-mesh sieve for analysis.

### Chemical analyses

For Hg, Cd, Pb, and Fe analyses, 0.20 g of sediment was weighed in a 55mL fluorocarbon (TFM) microwave vessel, 5 mL aqua regia was added, and less than 5 min was required for the completion of the specific reactions. The tubes were then tightly sealed. The mixture was then digested for 10 min using microwave heating in a suitable laboratory microwave unit (MARS 5, CEM). In this process, the temperature of each sample should rise to 180°C in less than 5.5 min and remain between 175°C and 185°C for the remaining 10 min irradiation period. After cooling, the content was diluted to 50 mL with Milli-Q water. The Hg content was determined using cold vapor atomic fluorescence spectrometry while the solution was limpid. The Cd, Pb, and Fe concentrations were measured using inductively coupled plasmamass spectrometry with a SCIEX Elan 9000(Perkin-Elmer, USA) equipped with a Ryton cross-flow nebulizer.

The sediment samples were air-dried, ground and passed through a 2-mm sieve. The pH was determined from the <2 mm fraction in a slurry of 1:2.5 sediment: water suspension using a calibrated ORION 1260 Ion Selective Electrode (Thermo Fisher Scientific, USA). The salt content was determined from the <2 mm fraction in a slurry of 1:5 sediment: water suspension using a conductivity meter. An aliquot of sediment (5 g wet weight) was stirred for 1 h with HCl 6 M under constant N_2_ flux, and the S^2−^ ions trapped in a 0.5 M NaOH solution were measured with methylene blue spectrophotometric method. Sodium acetate and conductance methods were used to determine the cation exchange capacity (CEC) and salt content of the samples. TN was determined using an elemental analyzer (Vario EL/MICRO cube, Elementar, Hanau, Germany). Sulfuric-perchloric acid digestion of sediments was used for TP determination, and TP was analyzed using a spectrophotometer (Tu-1810, Persee, China) through the Mo-Sb colorimetric method.

For determination of TOC and isotope composition, 5 g of sediment was weighed and soaked with 2 mol/L HCl to remove carbonate. The mixture was then washed using ultra-pure water to remove HCl, prior to freeze-drying and grinding. TOC was determined using an elemental analyzer (Vario EL/MICRO cube, Elementar, Hanau, Germany). δ^15^N and δ^13^C were determined using a Nu Horizon continuous-flow isotope ratio mass spectrometer (Nu Instruments, Wrexham, UK) and a perspective stable isotope ratio mass spectrometer (Nu Instruments). IAEA-CO-9 barium carbonate and IAEA-NO-3 potassium nitrate were used as standards for the calculations of δ^13^C and δ^15^N, respectively. Variations in ^15^N versus ^14^N, and ^13^C versus ^12^C were expressed as ‰ deviations relative to their reference standards in δ units, as follows:

$$\delta^{15}N(‰)=(\frac{R_{\mathrm{Sample}}}{R_{\mathrm{Standard}}}-1)\times 1000,$$ (1)

and;

$$\delta^{13}C(‰)=(\frac{R_{\mathrm{Sample}}}{R_{\mathrm{Standard}}}-1)\times 1000.$$ (2)

In these expressions, *R* is the corresponding ratio of either ^15^N/^14^N or ^13^C/^12^C.

### Quality control

In order to check analytical quality, sample preparation, and instrument performance, laboratory quality control was conducted via analyses of a certified reference sediment (i.e., sediment GBW-07310: 0.28±0.03, 1.12±0.08, 26±2, 720±90,271±23, and 400±40 mg/kg for total Hg, Cd, Pb, N, P, and TOC, respectively). All control samples were prepared and analyzed identically to the study samples. In this study, GBW-07310 reference total Hg, Cd, Pb, N, P, and TOC concentrations were 0.29±0.03, 1.20±0.09, 25±2.1, 643±72, 287±22, and 348±56 mg/kg respectively in three replicates.

### Statistical analysis

All data were analyzed using Microsoft Excel 2007 and SPSS version 12.0, and differences were considered statistically significant at *p*<0.05. All data in this study are shown as the mean±standard deviation(*n* = 3). Relationships between variables were based on Pearson’s correlation coefficients, and factor analysis was applied to group pollutants using SPSS 12.0. The statistical significance of observed differences between metals, TOC, and nutrients released in each sediment core was determined using ANOVA in SPSS 12.0. Mapping for each sample site was performed using ArcGIS 10.0 (ESRI).

### Metal accumulation assessment

Geochemical background concentrations of metals and NOAA (National Oceanic and Atmospheric Administration) sediment quality guidelines were applied to assess heavy metal accumulation and sediment pollution levels. The enrichment factor (*EF*), based on the normalization of a tested element against a conservative reference element, is widely used to estimate anthropogenic impacts on sediments. The geo-accumulation index (*I*_geo_) is another commonly applied index for assessing heavy metal contamination by comparing measured metal concentrations with local background levels [11]. In this study, the local background levels are the metal concentrations observed in preindustrial sediments. The *EF* and *I*_geo_ indices are defined as follows:

EF=(CnCRef)Sample(BnBRef)Background, (3)

and;

$$I_{\mathrm{geo}}=\log_{2}\left(\frac{C_{n}}{1.5B_{n}}\right).$$ (4)

In these equations, *C*_*n*_ is the measured metal concentration in the samples, *C*_Ref_ is the reference element concentration in the study environment, *B*_*n*_ is the geochemical background concentration of the metal and *B*_Ref_ is the reference element concentration in the reference environment. The value 1.5 in Eq 2 is a background matrix correction factor that takes into account possible variations in values resulting from lithogenic effects. A detailed explanation of *EF* and *I*_geo_ is presented in Abuduwailiet al. (2015)[11]. In the present study, Fe was used as the reference element for geochemical normalization. Background soil geochemical concentrations in Tianjin were reported as 0.05 mg/kg, 0.09 mg/kg, 20.32 mg/kg, and 3.49% for Hg, Cd, Pb, and Fe respectively, by Wu and Cao (2010)[12].

## Results and discussion

### Sediment characteristics

Surface sediment samples from the Beidagang Wetland Nature Reserve were separated into four fractions: clay (<2 μm, 17.1%), silt (2–20 μm, 21.9%), fine sand (20–200 μm, 45.3%), and coarse sand (>200 μm, 15.7%). The physicochemical sediment characteristics are summarized in Table 1. The average pH of the surface sediment samples was 7.8, lower than measured for the bottom sediments (pH = 8.1). The average TOC, TN, and TP concentrations were 1.38%, 0.115%, and 0.049%, respectively in the surface sediment samples. All nutrient concentrations were higher than measured in the bottom sediment samples (0.66%, 0.056%, and 0.021%). In addition, TOC/TN and δ^13^C values in the surface sediments were 10.66 and –21.6 ‰, respectively, which were lower than the corresponding values (12.13 and –20.5‰) in the bottom sediments. The TOC/TN ratio of organic matter is indicative of the source of organic matter; TOC/TN ratios exceeded 10 in the sediment core samples, which indicates the input of terrigenous organic matter [13].

**Table 1:** Physicochemical characteristics of sediment cores.

	Surface sediment(0–20 cm)	Bottom sediment(20–35 cm)
pH	7.8	8.1
Water content(%)	34.3	23.7
TOC(%)	1.38	0.66
TN(%)	0.115	0.056
TP(%)	0.049	0.021
TOC/TN	10.66	12.13
δ^13^C(‰)	-21.6	-20.5
δ^15^N(‰)	0.93	-0.02
Cl^−^(%)	0.11	0.06
Total salt content (%)	0.19	0.09
Hg(mg/kg)	0.114	0.088
Cd(mg/kg)	0.160	0.177
Pb(mg/kg)	30.0	23.8

The capacity of sediments to store and accumulate trace metals depends mainly on their CEC and adsorption capacity, which vary with clay content, types of clay minerals, organic matter, and oxide or hydroxide content. Results show that CEC in the surface sediment samples (13.4 meq/100g) was less than that in the bottom sediments (20.1 meq/100g), while δ^15^N in surface sediments (0.93‰) was greater than that in bottom sediments (–0.02‰). The total salt content in the surface sediment samples (1.91%) was greater than the corresponding value for bottom sediments (0.88%). The bottom sediments had the highest CEC values and adsorption capacity, while the surface sediments had the highest total salt content, which means higher mobility of Cd is expected due to higher total salt content.

### Trace metal accumulation and distribution

The concentrations of Hg, Cd, and Pb in the samples are shown in Table 1. Compared with the NOAA sediment quality guidelines [14], samples from the Beidagang Wetland Nature Reserve can be considered lowly polluted because they do not exceed the Effects Range-Low (ERL) values of 0.15 mg/kg, 1.2 mg/kg, and 46.7 mg/kg for Hg, Cd, and Pb, respectively. Metal concentrations in the sediment samples were also lower than the sediment quality guideline threshold (Threshold Effects Levels, TEL) and Probable Effects Levels (PEL) of 0.17 mg/kg and 0.486 mg/kg for Hg, 0.6 mg/kg and 3.53 mg/kg for Cd, and 35 mg/kg and 91.3 mg/kg for Pb, respectively. Measured values below these standard levels suggest that the health of wildlife is not at risk from trace metal contamination and show that sediment pollution is a minor concern in the Beidagang Wetland Nature Reserve.

Concentrations of Hg and Cd in surface sediments collected from the polluted rivers in Tianjin (the Beitang River, Haizhu River, Haihe River, and Dagu River) exceeded the ERL and TEL values and thus represent a health risk to wildlife. Domestic and industrial waste was directly discharged into these rivers, and high trace metal concentrations were derived from sewage and wastewater effluents [15–17]. These data show that the concentrations of Hg and Cd in the Beidagang Wetland Nature Reserve are relatively low.

The Beidagang Wetland Nature Reserve is becoming more saline by the combined effects of sea level rise and altered hydrological and climatic conditions [9,10]. The ions of Ca^2+^, Mg^2+^, and Cl^−^ were also measured in the surface sediments at concentrations as high as 0.35, 0.82, and 1.09g/kg, respectively. Hg and Cd can easily combine with Cl^−^ ion to form HgCl_4_^2−^ and CdCl_2_. The stability and solubility of these complexes were higher than the affinity of Hg and Cd for the solid sediment phase. Furthermore, substantial amounts of Hg, Cd, and Pb associated with the dissolved fraction could also be easily leached from the sediments under the attack of ions such as Ca^2+^ and Mg^2+^. These might increase the mobility of heavy metals and would result in the metals finally being lost through water induced leaching.

**Fig 2:**
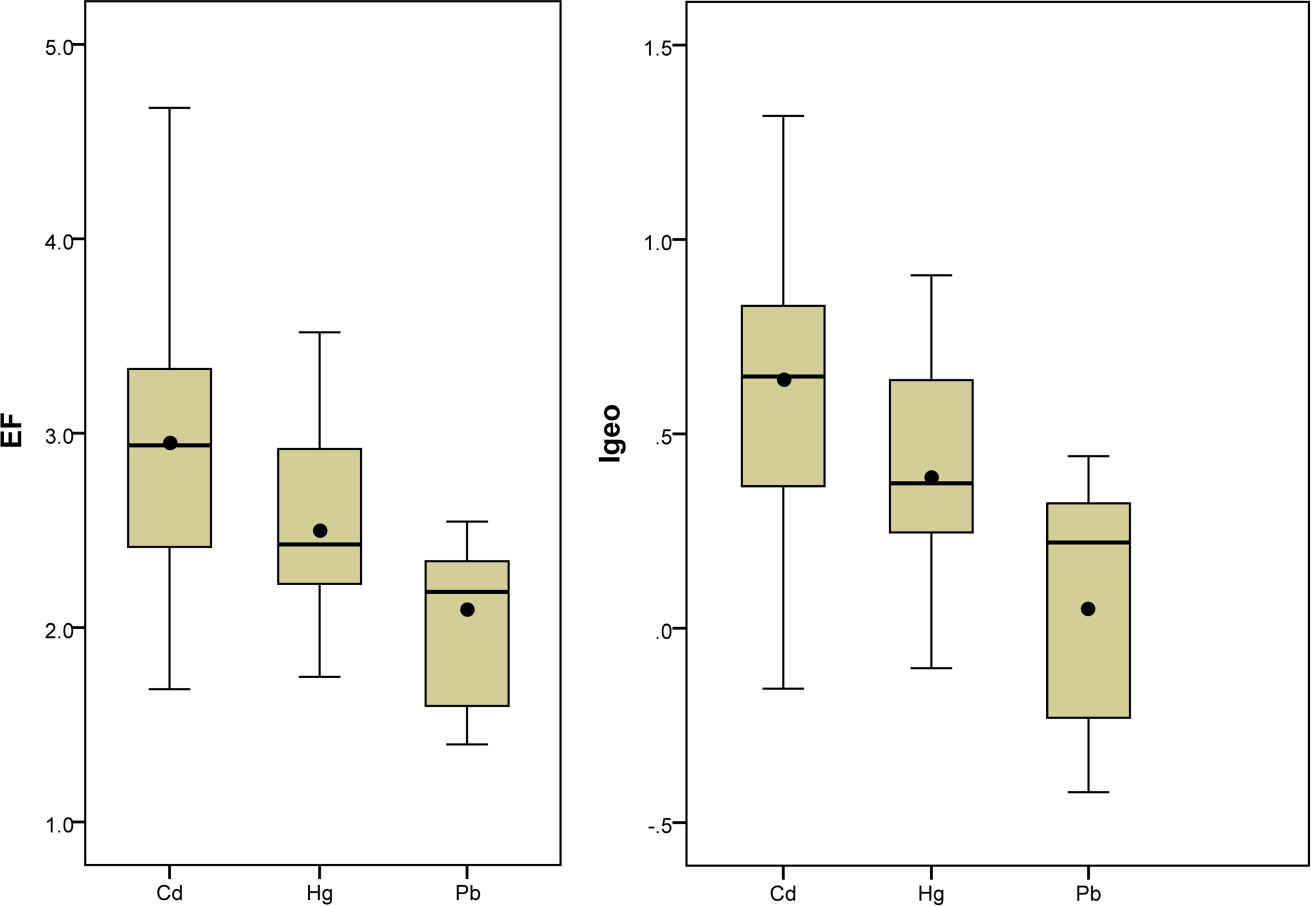
Sediment core trace metal *EF* and *I*_geo_ results from the Beidagang Wetland Nature Reserve. The box extends from the lower quartile (25%) to the upper quartile (75%), covering the median (line) and the mean (black point) values; Vertical bars represent the error.

Enrichment factor and *I*_geo_ index results for sediment trace metals from the Beidagang Wetland Nature Reserve are presented in Fig 2. These data show that overall the sediment cores from different sites have similar *EF* and *I*_geo_ values for Hg, Cd, and Pb. The average *EF* values for Cd, Hg, and Pb were 2.98, 2.54, and 2.03, respectively, which indicates moderate contamination with these metals (2≤*EF*<5). Average *I*_geo_ values for Cd, Hg, and Pb were 0.65, 0.42, and 0.09, respectively, which indicates that sediments in the reserve range from uncontaminated to moderately contaminated (0<*I*_geo_≤1). Tang et al (2013) found that Cd, Cr, Cu, and Pb concentrations in Beijing urban street dust were elevated in comparison with Beijing soil background values. In this study, *I*_geo_ levels ranging from 0 to 5 were found with about 80% of samples below the moderately polluted level [18]. Trace metal pollution universally occurred in the soil, street dust, and sediment in Beijing-Tianjin-Hebei region [4,8,12].

**Fig 3:**
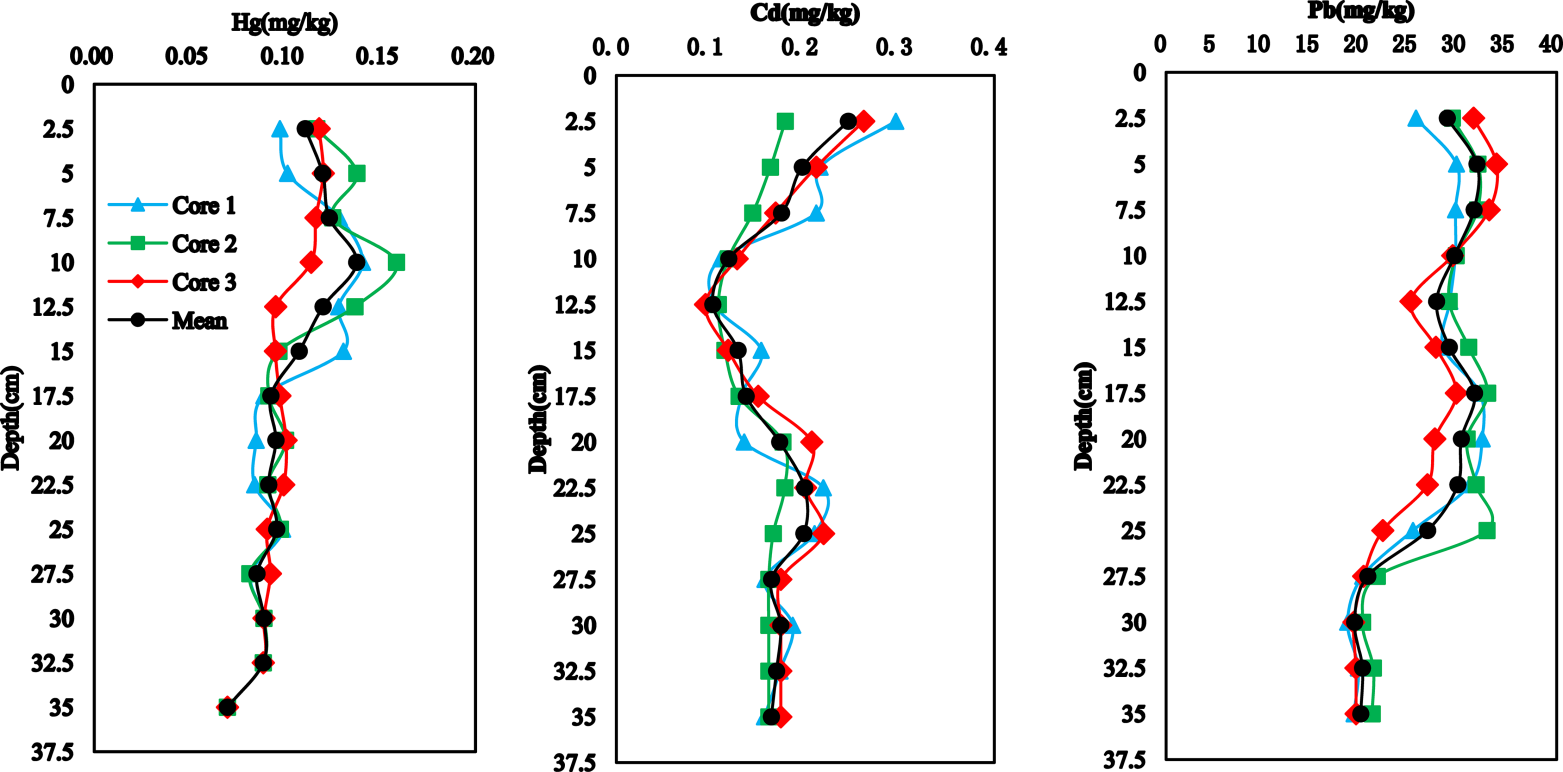
Vertical distributions of trace metals in sediment cores.

Vertical profiles of Hg, Cd, and Pb are shown in Fig 3. The average concentration of Hg was 0.07 mg/kg at approximately 35 cm depth, close to the sediment geochemical background concentration (0.05 mg/kg) for Hg in Bohai Bay. The concentration profile of the three sediment cores shows an increase in Hg concentration from the base of the profile to a height of approximately 17.5 cm, followed by a fairly uniform distribution from a depth of approximately 15 cm to the surface. The Hg concentration at a depth of approximately 10 cm was 0.14 mg/kg. Sediment profile results also show that Cd concentrations decrease from the surface to a depth of approximately 12.5 cm, increase from 15 cm to about 25 cm, and remain fairly uniform in distribution from about 27.5 cm to the bottom. The average Cd concentration (0.177 mg/kg) in bottom sediments (0–20 cm) was greater than the corresponding value (0.160 mg/kg) for surface sediments (0–20 cm). Previous studies showed that an increase in salinity caused by any salt promoted a higher and faster release of Cd in sediments than other metals (Pb, Cu, and Zn). The main mechanism regulating Cd mobility was the formation of Cd-chlorides [19,20]. Concentrations of Cd were 0.17 mg/kg at approximately 35 cm depth, close to the sediment geochemical background value (0.14 mg/kg) for Cd in Bohai Bay. Finally, profiles show that Pb concentrations decrease from the top to approximately 12.5 cm depth, then remain fairly uniform in distribution across the profile. Lead concentrations were equal to 20.7 mg/kg at approximately 35 cm, again close to the sediment geochemical background value (16.6 mg/kg) for Pb in Bohai Bay. A previous study reported Pb contents in a Bohai Sea sediment cores (0–35 cm) ranging between 23.65 mg/kg and 28.33 mg/kg (mean value: 25.14 mg/kg) [21].

There was no statistically significant difference between the concentrations of Hg, Cd, and Pb measured in cores 1, 2, and 3 (ANOVA, *P*>0.05). There were obvious temporal variations but no significant spatial differences in trace metal concentrations of core sediments in the Beidagang Wetland Nature Reserve.

### Sources of carbon and nitrogen

**Fig 4:**
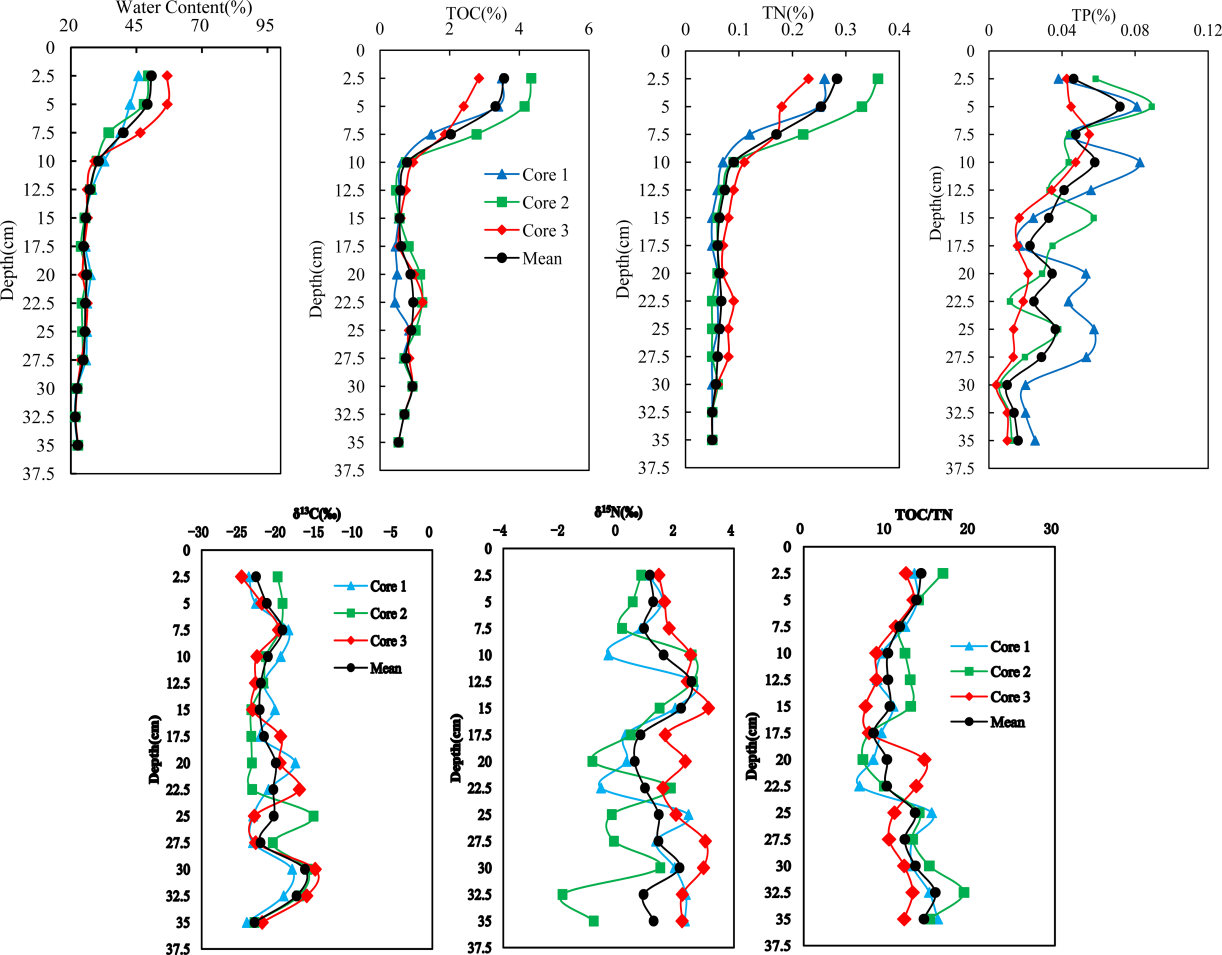
Vertical distribution profiles of water content, TOC, TN, TP, δ^13^C, δ^15^N, and TOC/TN.

Sediment profiles of water content, TOC, TN, and TP are shown in Fig 4. All results show fairly uniform distributions (mean values of 46.6%, 2.97%, 0.19%, and 0.055%, respectively) from the surface to a depth of approximately 7.5 cm. Below 7.5 cm there is a sudden and abrupt change, with values decreasing by more than half (mean values of 25.2%, 0.74%, 0.08%, and 0.029%, respectively). There was no statistically significant difference between the water content, TOC, TN, or TP measured in cores 1, 2, and 3 (ANOVA, *P*>0.05).

Sediment profiles for TOC/TN, δ^13^C, and δ^15^N are also shown in Fig 4. There is a significant difference in theδ^15^N values between the sediment cores (ANOVA, *P*<0.05), while the TOC/TN ratios and δ^13^C values show no significant difference. Elemental ratios such as TOC/TN are commonly used to indicate the biodegradability of dissolved organic matter [22,23]. These results indicate that TOC is similarly degraded in each core, and TOC and TN did not originate from sewage discharge.

Previous studies have shown that the TOC/TN ratio for fresh algal organic matter is between 8 and 3 and that the ratio for terrigenous organic matter from vascular higher plants is around 20 or higher[24]. Thus, a TOC/TN ratio in sediment organic matter (SOM) greater than 8 is thought to be indicative of influence from these two sources. If the TOC in terrigenous SOM accounts for a higher proportion, the TOC/TN ratio will be even higher. As a result, residues and decaying matter of aquatic plants (e.g., algae, reeds, and Typha) are considered the two main sources of SOM in the Beidagang Wetland Nature Reserve. The TOC/TN values in this study range from 6.66 to 22.60 (average: 12.00±3.62) and are significantly higher than those calculated in a previous study (range: 6.73 to 9.78; average: 7.73±0.750) of the Yongdingxin River estuary (ANOVA, p = 0.001) [25]. Thus, higher TOC/TN values may reflect lower anthropogenic inputs in the Beidagang Wetland Nature Reserve compared to the Yongdingxin River estuary.

As shown in Figs 3 and 4, the profiles of TOC and TN are significantly different to the profiles of Hg, Cd, and Pb. Variations in Hg and Cd concentration in sediment cores were also different, and results show that the trace metals (Hg, Cd, and Pb), TOC, and nutrients do not exhibit either common behaviors or sources in the sediments. The Hg and Cd concentration profiles in the three sediment cores show a slow increase from the bottom to the top, implying that the concentrations at the base of cores may be natural values (i.e., geochemical background concentrations).

**Fig 5:**
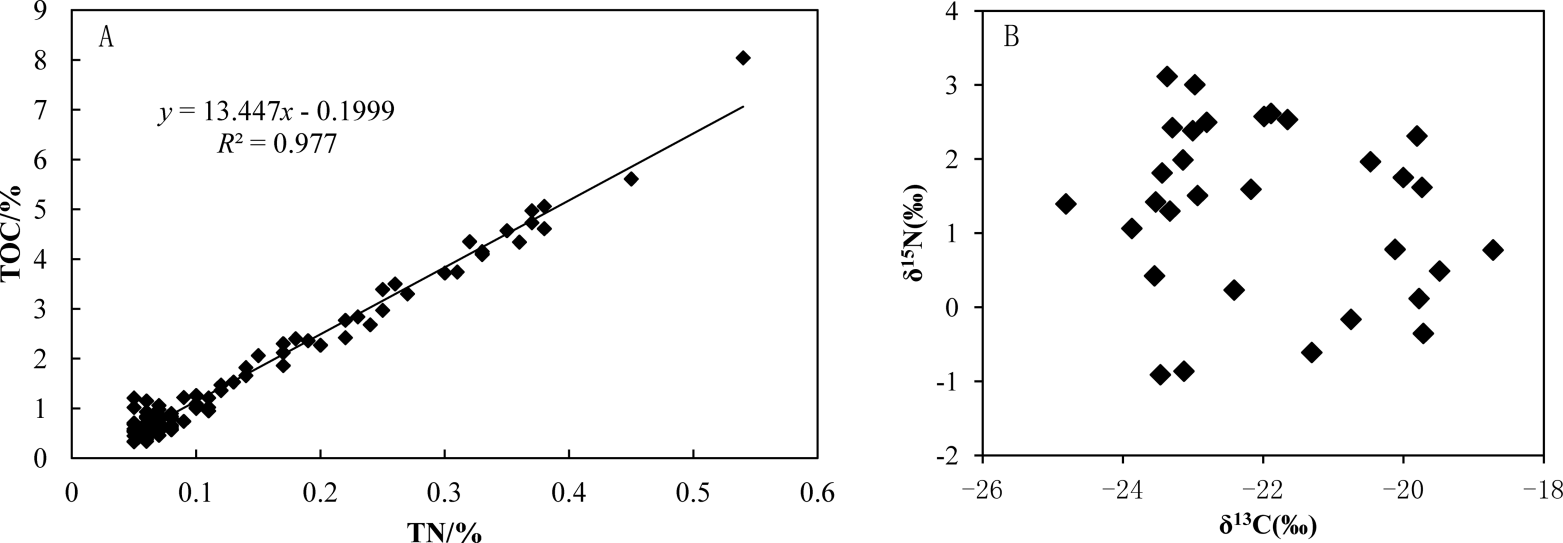
(A) TOC concentration compared to TN concentration; (B) δ^15^N compared toδ^13^C.

Fig 5 shows the TOC concentration compared to TN, and δ^15^N compared toδ^13^C.These data show that TOC and TN are significantly correlated (*p*<0.01), indicating that they share common behaviors or sources (Fig 5A). Bulk sedimentary stable isotopes of organic carbon (δ^13^C) and TOC/TN ratios have been widely and successfully used as markers to estimate changes in the relative proportions of terrigenous and marine organic matter in coastal sediments [26].

**Fig 6:**
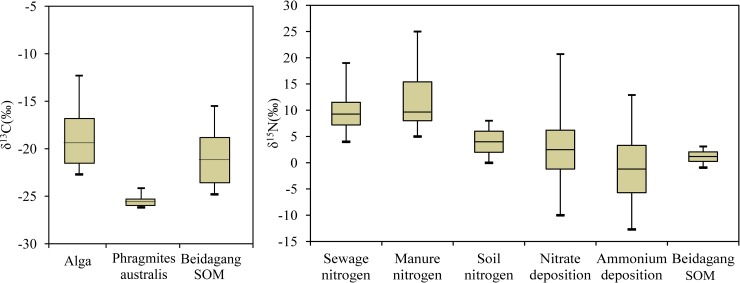
Box plots of δ^13^C and δ^15^N values from various sources. The lower and upper edges of the box represent 25^th^ and 75^th^ percentiles, encompassing the mean value (line); the lower and upper bars represent 10^th^ and 90^th^ percentiles. (Data from the references [28–30]; data in Beidagang SOM from the present study).

Fig 5B shows that δ^13^C values for sediments in the Beidagang Wetland Nature Reserve ranged from –24.8‰ to –18.7‰ (average: –21.2±2.38‰). Box plots of δ^13^C and δ^15^N values from various sources are shown in Fig 6. Marine organic matter is typically enriched in ^13^C compared to terrestrial plant material because of differences in photosynthetic mechanisms and carbon sources. Terrestrial C3 vascular plants and their C4 counterparts employ different photosynthetic pathways and thus produce different δ^13^C values. A δ^13^Cvalue in the range of C3 (–28‰to–26‰) and C4 plants (–14‰to–12‰) as well as a C/N ratio greater than 12 indicates a terrestrial end member, while a δ^13^C in the range –22‰to –19‰ and a C/N ratio less than 8 suggests an aquatic end member[27].Therefore, results indicate that TOC in the study sediments is not predominantly derived from terrestrial and soil organic matter. Potential alternative sources include residues and decaying matter of aquatic plants (e.g., algae, reed, and Typha).

Results show that δ^15^N values for sediments in the Beidagang Wetland Nature Reserve range from –0.911‰ to 3.11‰ (Fig 5B). Increases in reactive nitrogen are attributed to intensive land use, the increased use of N-containing organic and inorganic fertilizers, animal manure, discharge of human sewage, and elevated atmospheric N deposition[31–33]. Organic and inorganic fertilizers (i.e., ammonium, NO_3_^–^, and urea) have typical δ^15^N values between –6‰ and +6‰, while typicalδ^15^N values for atmospheric N deposition are between –13‰ and +13‰. Variation within this range is controlled by complex chemical reactions in the atmosphere as well as a variety of anthropogenic sources including fossil fuel combustion. Manure and sewage are enriched in δ^15^N relative to other N sources; δ^15^Nvalues that originate from manure are between +5‰ and+25‰, while those from sewage are between +4‰ and +19‰. Typical δ^15^N values for soil nitrogen range between 0‰ and+8‰ (Fig 6). Results show that TN in the Beidagang Wetland Nature Reserve sediments mainly originates from soil nitrogen and elevated atmospheric nitrogen deposition. Thus, fertilizers, sewage, and manure were not the main sources of nitrogen in this system, indicating that sewage and wastewater do not discharge into the nature reserve.

Results show that δ^13^C values in the Beidagang Wetland Nature Reserve sediments range from –24.8 ‰ to –18.7‰ (average: –21.2±2.38‰), significantly higher than the range –23.7 ‰ to –22.5‰ previously reported for the Yongdingxin River estuary sediments (average: –23.1±0.35‰) (ANOVA, *p* = 0.001)[25]. If δ^13^C values are in the range –22‰ to –16‰, organic matter could be from a mixture of terrigenous and aquatic components. One possible explanation for this result is that phytoplankton prefers to absorb CO_2_ in ^12^C enriched air which results in a decreased δ^13^C value. In contrast, the δ^15^N values in this study ranged from –0.911‰ to 3.11‰ (average: 1.16±1.24‰), significantly less than the range (–2.53‰ to 6.77‰, average: 2.28±2.11‰) reported for Yongdingxin River estuary sediments (ANOVA, *p* = 0.004) and suggesting a slightly different enrichment of N in these samples. The low δ^15^N reported in the nature reserve sediments might be due to contributions from soil nitrogen, as terrestrial plant ecosystems typically have lower δ^15^N. Lower δ^15^N values may also reflect lower anthropogenic input of nitrogen into the Beidagang Wetland Nature Reserve compared with the Yongdingxin River estuary. These results show that the sedimentary environment of the Beidagang Wetland Nature Reserve has not been seriously impacted by human activities such as the discharge of wastewater and sewage. Thus, the accumulation of trace metals has likely been the result of atmospheric deposition.

### Multivariate statistical analysis

**Fig 7:**
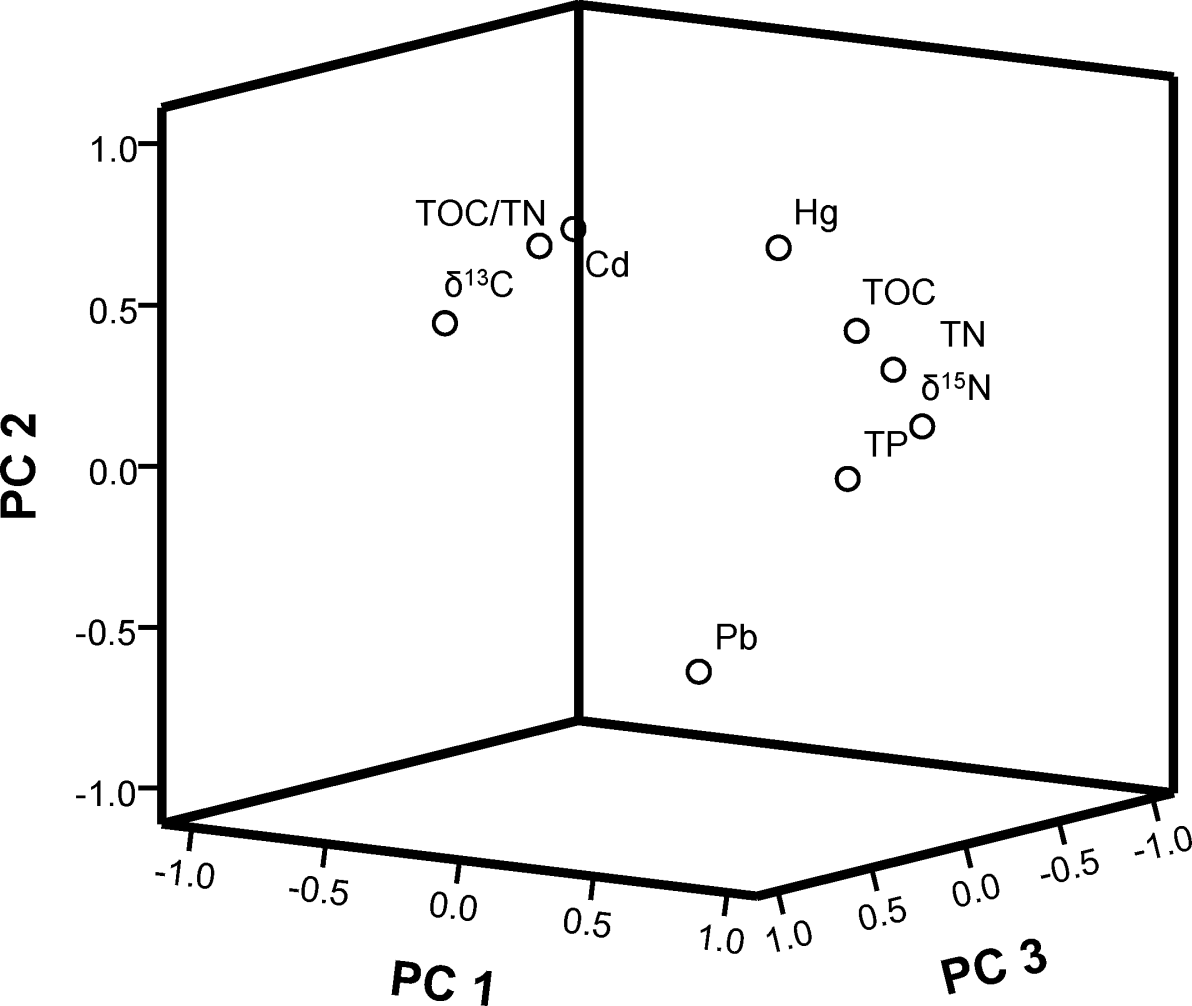
Loading plots for the first three PCs.

Major sources of trace metal pollution in aquatic ecosystems are domestic wastewater effluents, coal-burning power plants, nonferrous metal smelters, iron and steel plants, and dumping of sewage sludge. The atmosphere is the major route for Pb entering natural waters [34]. Principal component analysis (PCA) was performed to further identify statistically significant sources influencing trace metal contamination in the Beidagang Wetland Nature Reserve. For this analysis, only PCs with eigenvalues greater than 1 were considered important; the analysis revealed that three factors were responsible for 71.55% of the total variation. The PCA results, with variable loadings, variance, and PCs, are presented in Table 2 and Fig 7.

**Table 2:** Loadings of measured variables in the Beidagang Wetland Nature Reserve, northern China.

Parameters	PC1	PC2	PC3
TOC(%)	**0.81**	0.47	0.14
TN(%)	**0.88**	0.37	0.05
TP(%)	**0.80**	0.02	0.19
TOC/TN	-0.21	**0.67**	0.38
δ^13^C(‰)	-0.51	0.41	0.47
δ^15^N(‰)	**0.50**	0.03	-0.64
Hg(mg/kg)	0.33	**0.65**	-0.12
Cd(mg/kg)	-0.54	**0.59**	-0.26
Pb(mg/kg)	0.46	-0.57	**0.50**
Eigenvalue	3.22	2.06	1.16
%Total variance	35.83	22.84	12.88
Cumulative total variance	35.83	58.67	71.55

The results show that component 1 (PC1) has a total variance of 35.83% and is substantially positively loaded with TOC, TN, TP, and δ^15^N. All four of these variables are generated from the residue and decaying organic matter of aquatic plants. The δ^15^N value in plants is close to in the values resulting from atmospheric deposition and soils. In contrast, PC2 is responsible for 22.84% of the total variation, and a moderate amount of Hg and Cd is loaded into the sediments. This is significant because atmospheric deposition is an important source of Hg and Cd[8].

The trace metals Hg and Cd are significant pollutants that influence soil quality in Beijing and Tianjin. Previous research has demonstrated that coal burning and weathering of HgS, used as a pigment in ancient buildings, significantly contributes to higher Hg concentrations [35]. Coal combustion has been identified as the major source of particulate mercury in aerosol samples in Beijing [6]. Because there is a potential link between atmospheric particles and contaminated soils, Schleicher et al. (2016) compared atmospheric particulate Hg concentrations in total suspended particulates samples from Beijing with soil guideline values for the concentration of Hg [36]. Their results showed that average particulate Hg concentrations in August were 1.03±0.21mg/kg between 2006 and 2010, 2.24±0.85mg/kg in December between 2005 and 2010, and 2.11±0.66 mg/kg in January between 2006 and 2011. These values are of the same magnitude as the soil guideline value of 1.00 mg/kg Hg for residential areas in the UK, and the Chinese Grade II guideline.

Results show that PC3 has a total variance of 12.88% and is responsible for the positive loading of Pb into the Beidagang Wetland Nature Reserve sediments. Lead mainly originates from traffic contaminants, and therefore Pb is likely to have come from anthropogenic sources including vehicular traffic and industrial discharge, as reported in previous work on soils in Tianjin [8]. In another related study, Shi et al. (2008) identified the source of metals in urban dust using geostatistical and multivariate analyses, reporting that Pb mainly originates from traffic contamination and Cd largely comes from point-source industrial pollution [37]. Levels of Pb and Cd in urban dust were higher than in suburban dust, while concentrations of Hg were higher in suburban dust, indicating a different main source [38].

**Fig 8:**
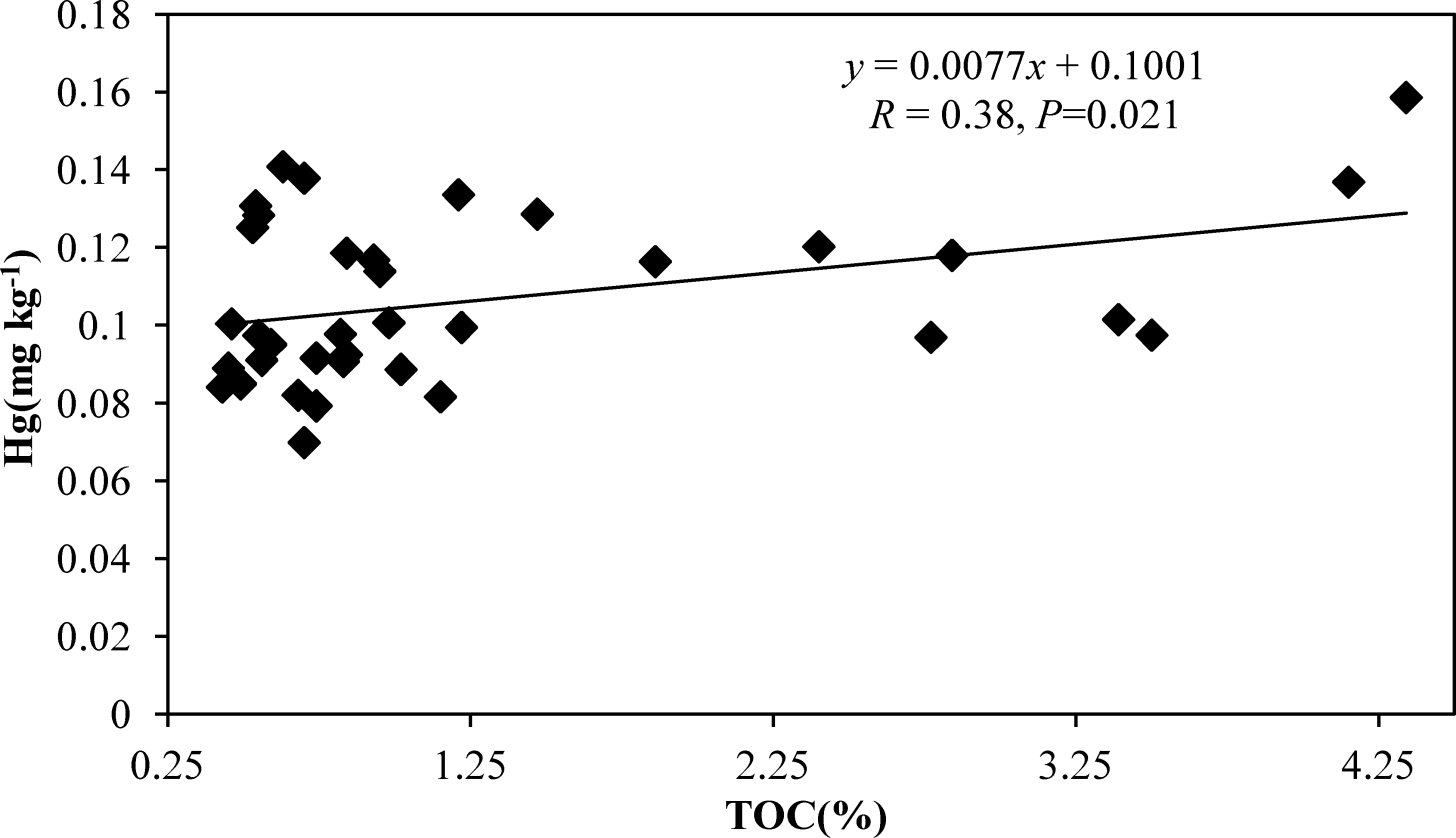
Hg concentrations compared to TOC concentrations.

Pearson's correlation analysis has often been employed to determine the common source (and/or carrier substances) of trace metals. Thus, in the present study, a correlation matrix was calculated for the elements in question to discuss sediment responses to anthropogenic contaminants in the Beidagang Wetland Nature Reserve (Table 3). Results show that TOC is positively correlated with TN, TP, and Hg, while TN is significantly correlated with TP, δ^15^N, and Hg. Fe is positively correlated with Pb and TP. Results also indicate that TOC, TN, and TP may have common sources. A positive linear correlation was observed between TOC and Hg (*r* = 0.38, *P =* 0.021, *n* = 51) (Fig 8), while Cd was not significantly correlated with TOC. Results also indicate that Hg and Cd have different behaviors and sources. Divalent metals such as Cd and Pb have a high affinity with sulfide in anoxic sediment, while sulfide is not correlated with Hg, Cd, and Pb in the Beidagang Wetland Nature Reserve. The sediments were aerobic due to the lack of water resources and the low water level in the wetland. Sulfide might become oxidized to sulfate. A significant negative correlation (*r* = -0.63, *p*<0.01) between Cd and Pb also shows that these metals have different behaviors. However, a positive correlation between these trace metals in contaminated sediments of western Bohai Bay and in adjacent estuaries have been reported in previous studies [39,40]. Correlations were significant in rivers and estuaries affected by human activities while the correlations were poor in the Beidagang Wetland Nature Reserve.

**Table 3:** Correlation coefficients for TOC, TN, TP, TOC/TN, δ^15^N, δ^13^C, Hg, Cd, and Pb.

	TOC	TN	TP	Sulfide	TOC/TN	δ^13^C	δ^15^N	Fe	Hg	Cd	Pb
TOC	1	0.97^a^	0.58^a^	0.32	0.24	-0.22	0.28	-0.29	0.38^b^	-0.16	0.13
TN		1	0.62^a^	0.15	0.02	-0.32	0.34^b^	0.16	0.38^b^	-0.23	0.19
TP			1	0.41	-0.15	-0.32	0.21	0.37^b^	0.30	-0.38^b^	0.38^b^
Sulfide				1	0.25	-0.29	0.26	-0.20	0.02	0.19	0.22
TOC/TN					1	0.39^b^	-0.18	-0.32	0.12	0.31	-0.31
δ^13^C						1	-0.34^b^	0.09	0.21	0.29	-0.17
δ^15^N							1	0.27	0.28	-0.23	0.01
Fe								1	0.09	0.26	0.43^b^
Hg									1	0.18	-0.13
Cd										1	-0.63^a^
P^b^											1

^a^ Correlation is significant at the 0.01 level (2-tailed).

^b^ Correlation is significant at the 0.05 level (2-tailed).

Previous studies have shown that Hg in sediments in polluted rivers and estuaries in Tianjin are significantly correlated with TOC, and not positively correlated with Cd [16,40]. The total Hg concentrations in the Haihe River and Dagu Drainage River sediments were significantly correlated with TOC contents (*r* = 0.87, *p* = 0.99, *n* = 21) [16]. This result is also in agreement with a previous study on Haihe River sediments; a strong correlation between Hg and TOC concentrations was found in the heavily polluted river sediments, indicating that concentrations of Hg were significantly influenced by sediment TOC contents [15]. A recent investigation indicated that Hg was significantly correlated with TOC and the correlation explained 78% of the variance in the data; this investigation showed that Hg was associated with organic matter in water bodies [41]. All these results suggest that sediment organic matter plays an important role in the sedimentary accumulation of Hg in wetland ecosystems. Although organic matter plays the most important role in the distribution of trace metals in sediments, its role is not well understood [23]. For example, Sanei and Goodarzi reported that the strong affinity between Hg and soluble organic matter is due not only to its chemical reactivity but also to the physical characteristics of these labile compounds [42].

### Atmospheric wet or dry deposition

The Beijing-Tianjin-Hebei region has a very large consumption of energy and power, the majority of which is sourced from coal combustion, identified as the major source of particulate Hg in Beijing and Tianjin [5,6].Total global Hg emissions from anthropogenic sources to the atmosphere range from 1900 to 2900 t/yr; East Asia currently accounts for almost 40% of total global anthropogenic emissions of Hg [43].Total anthropogenic Hg emissions in China were estimated to have continuously increased from 356 t in 2000 to 538 t in 2010 with an average annual increase rate of 4.2% [44]. Coal-fired power plants in China emit less than 100 tons Hg directly to the atmosphere every year due to nationwide air control actions [45]. The Beijing, Tianjin, and Hebei provinces combusted 23, 53, and 302 million tons of coal in 2013, respectively, and total coal combustion was estimated to be 378 million tons/yr in this region. Coal combustion from coal-fired power plants was 13.37, 24.2, and 89.58 million tons/yr in these provinces, respectively in 2013[46]. The emission factors for Hg, Cd and Pb vary in the ranges of 20–430 mg/ton coal, 1.25–13.11 mg/ton coal, and 307.39–2965.73 mg/ton coal, respectively [47,48]. Atmospheric trace metals from coal combustion are deposited on the ground and on vegetation before being brought into water circulation. Atmospheric emissions represent a major pathway for trace metals to enter the surface environment. Wet and dry deposition of atmospheric Hg is important sources of Hg to terrestrial ecosystems in the nature reserve.

In recent decades, there has been increasing research interest in air pollutants in the Beijing-Tianjin-Hebei region. However, only a few studies have considered the atmospheric deposition of trace metals. The particulate Hg concentration in aerosol samples from Beijing between January and December 2006 was 573±551 ng/m^3^ [4]. The Hg concentrations in snow samples from Tianjin in three heating seasons in winter (2012–2015) ranged from 0.06 to 0.23 μg/L, and the Cd concentrations ranged from 0.15 to 0.72 μg/L [49]. Wet deposition of Hg in Beijing was 101.52 μg/(m^2 ^yr) (Nov 1994–Nov 1995) and 123.09 μg/(m^2 ^yr) (Nov 1995–Nov 1996) [50]. The atmospheric dry deposition fluxes of Hg and Cd in Tianjin were 35.3–37.5 μg/(m^2 ^yr) and 97.4–103.6μg/(m^2^yr), respectively [5]. Atmospheric deposition of Hg in the Bohai Sea was 31.0 μg/(m^2^ yr) [51]. The atmospheric wet depositions of Pb in Mount Tai and Jiaozhou Bay, northern China were 7570 and 2210 μg/(m^2^yr), respectively, while the depositions of Cd were 340 and 130 μg/(m^2^yr), respectively [52]. A previous study reported that the annual flux of atmospheric Hg deposition in China’s adjacent seas was 140 tons. The proportion of Hg that was deposited into sediment was 50% of the total Hg output. Hg evasion from surface sediment and surface water comprised 2.4% of the total Hg input and 26% of the total Hg output of the seas [51]. Industrial and urban areas have high anthropogenic emissions of Hg and are regarded as Hg output areas. In contrast, terrestrial ecosystems in nature reserves, which have much lower Hg emissions and are exposed to high Hg loadings from polluted areas, are sinks of atmospheric Hg and experience Hg accumulation.

The concentrations of Hg and Cd in topsoil from Tianjin in 2013 were 0.40 μg/g and 0.18 μg/g, respectively [8], while Hg and Cd concentrations in topsoil from Beijing in 2015 were 0.40 μg/g and 0.72 μg/g, respectively [35,53]. The concentrations were of the same magnitude as the values in the Tianjin and Beijing topsoil. These concentrations were much higher than the Tianjin soil background values, 0.05 μg/g for Hg and 0.09 μg/g for Cd. Atmospheric deposition was identified as an important source of trace metals, especially Hg and Cd, in soils and the ecosystem in the Beijing-Tianjin-Hebei region.

## Conclusions

Combined analysis of trace metals, and stable carbon and nitrogen isotopic composition of organic matter was used to assess the origin and accumulation in sediments from the Beidagang Wetland Nature Reserve. Based on the enrichment factor and the geo-accumulation index calculated in the present study, the sediments can be considered to be unpolluted to moderately polluted with Hg, Cd, and Pb. However, compared to other metal-polluted rivers in Tianjin, concentrations of Hg and Cd in the sediments were relatively low. There were obvious temporal variations but no significant spatial differences in trace metal concentrations in the sediment cores. Stable isotopes of carbon and nitrogen indicate that urban sewage and industrial waste do not discharge into the Beidagang Wetland Nature Reserve, and thus the potential sources of TOC in this area are residue and decaying aquatic plant matter (e.g., algae, reed, and Typha). TN mainly originates from the soil and elevated atmospheric nitrogen deposition. Atmospheric deposition is the main source of trace metals in sediments from the Beidagang Wetland Nature Reserve. Trace metal concentrations should be monitored to provide basic information about the impact of atmospheric deposition in Tianjin. Our results provide a baseline dataset for trace metal accumulation predating the industrialization of Tianjin to the present day for key locations. This can be used for comparisons with other regions to assess the effects of atmospheric deposition on trace metal contamination in northern China.

## Supporting information

S1 Data Complete primary data file. (DOCX)
