# Supplementary material for: Trace metals, organic carbon and nutrients in the Beidagang Wetland Nature Reserve, northern China
Source: PLoS One. 2018 Oct 2;13(10):e0204812. doi: 10.1371/journal.pone.0204812 (PMC6168145; doi:10.1371/journal.pone.0204812)
Supplement: S1 Data — (DOCX) [file pone.0204812.s001.docx]

**S1 Table.** Hg, Cd, and Pb contents in sediments from the Beidagang Wetland Nature Reserve.

| Depth(cm) | Hg (mg/kg) | | | | Cd (mg/kg) | | | | Pb (mg/kg) | | | |
| --- | --- | --- | --- | --- | --- | --- | --- | --- | --- | --- | --- | --- |
|  | Core 1 | Core 2 | Core 3 | Mean | Core 1 | Core 2 | Core 3 | Mean | Core 1 | Core 2 | Core 3 | Mean |
| 2.5 | 0.10 | 0.12 | 0.12 | 0.11 | 0.18 | 0.30 | 0.26 | 0.25 | 29.3 | 25.6 | 31.5 | 28.8 |
| 5 | 0.10 | 0.14 | 0.12 | 0.12 | 0.16 | 0.22 | 0.21 | 0.20 | 31.9 | 29.7 | 33.8 | 31.8 |
| 7.5 | 0.13 | 0.13 | 0.12 | 0.12 | 0.14 | 0.21 | 0.17 | 0.17 | 31.9 | 29.6 | 33.1 | 31.5 |
| 10 | 0.14 | 0.16 | 0.11 | 0.14 | 0.12 | 0.11 | 0.13 | 0.12 | 29.7 | 29.6 | 29.3 | 29.6 |
| 12.5 | 0.13 | 0.14 | 0.10 | 0.12 | 0.11 | 0.10 | 0.09 | 0.10 | 29.0 | 29.0 | 25.1 | 27.7 |
| 15 | 0.13 | 0.10 | 0.10 | 0.11 | 0.11 | 0.15 | 0.12 | 0.13 | 31.0 | 28.4 | 27.6 | 29.0 |
| 17.5 | 0.09 | 0.09 | 0.10 | 0.09 | 0.13 | 0.13 | 0.15 | 0.14 | 32.9 | 32.1 | 29.7 | 31.6 |
| 20 | 0.08 | 0.10 | 0.10 | 0.10 | 0.18 | 0.14 | 0.21 | 0.17 | 30.8 | 32.4 | 27.5 | 30.2 |
| 22.5 | 0.08 | 0.09 | 0.10 | 0.09 | 0.18 | 0.22 | 0.20 | 0.20 | 31.7 | 31.1 | 26.8 | 29.9 |
| 25 | 0.10 | 0.10 | 0.09 | 0.10 | 0.17 | 0.21 | 0.22 | 0.20 | 32.9 | 25.3 | 22.2 | 26.8 |
| 27.5 | 0.08 | 0.08 | 0.09 | 0.09 | 0.16 | 0.16 | 0.17 | 0.16 | 21.6 | 20.1 | 20.2 | 20.7 |
| 30 | 0.09 | 0.09 | 0.09 | 0.09 | 0.16 | 0.19 | 0.17 | 0.17 | 20.1 | 18.6 | 19.2 | 19.3 |
| 32.5 | 0.09 | 0.09 | 0.09 | 0.09 | 0.16 | 0.17 | 0.17 | 0.17 | 21.2 | 19.7 | 19.4 | 20.1 |
| 35 | 0.07 | 0.07 | 0.07 | 0.07 | 0.16 | 0.16 | 0.17 | 0.16 | 21.1 | 19.2 | 19.5 | 19.9 |

**S2 Table.** TOC, TN, and TP contents in sediments from the Beidagang Wetland Nature Reserve.

| Depth(cm) | TOC (%) | | | | TN (%) | | | | TP (%) | | | |
| --- | --- | --- | --- | --- | --- | --- | --- | --- | --- | --- | --- | --- |
|  | Core 1 | Core 2 | Core 3 | Mean | Core 1 | Core 2 | Core 3 | Mean | Core 1 | Core 2 | Core 3 | Mean |
| 2.5 | 3.50 | 4.34 | 2.84 | 3.56 | 0.26 | 0.36 | 0.23 | 0.28 | 0.04 | 0.06 | 0.04 | 0.05 |
| 5 | 3.39 | 4.15 | 2.40 | 3.31 | 0.25 | 0.33 | 0.18 | 0.25 | 0.08 | 0.09 | 0.04 | 0.07 |
| 7.5 | 1.47 | 2.77 | 1.86 | 2.03 | 0.12 | 0.22 | 0.17 | 0.17 | 0.04 | 0.04 | 0.05 | 0.05 |
| 10 | 0.63 | 0.74 | 0.95 | 0.77 | 0.07 | 0.09 | 0.11 | 0.09 | 0.08 | 0.04 | 0.05 | 0.06 |
| 12.5 | 0.55 | 0.46 | 0.74 | 0.58 | 0.06 | 0.07 | 0.09 | 0.07 | 0.06 | 0.03 | 0.03 | 0.04 |
| 15 | 0.54 | 0.56 | 0.59 | 0.56 | 0.05 | 0.06 | 0.08 | 0.06 | 0.02 | 0.06 | 0.02 | 0.03 |
| 17.5 | 0.45 | 0.82 | 0.55 | 0.61 | 0.05 | 0.06 | 0.07 | 0.06 | 0.02 | 0.03 | 0.02 | 0.02 |
| 20 | 0.49 | 1.15 | 0.98 | 0.87 | 0.06 | 0.06 | 0.07 | 0.06 | 0.05 | 0.03 | 0.02 | 0.03 |
| 22.5 | 0.43 | 1.21 | 1.22 | 0.95 | 0.06 | 0.05 | 0.09 | 0.07 | 0.04 | 0.01 | 0.02 | 0.02 |
| 25 | 0.84 | 1.02 | 0.83 | 0.90 | 0.06 | 0.05 | 0.08 | 0.06 | 0.06 | 0.04 | 0.01 | 0.04 |
| 27.5 | 0.68 | 0.70 | 0.84 | 0.74 | 0.05 | 0.05 | 0.08 | 0.06 | 0.05 | 0.02 | 0.01 | 0.03 |
| 30 | 0.93 | 0.93 | 0.93 | 0.93 | *0.05* | 0.06 | 0.06 | 0.06 | 0.02 | 0.01 | 0.00 | 0.01 |
| 32.5 | 0.70 | 0.70 | 0.70 | 0.70 | *0.05* | 0.05 | 0.05 | 0.05 | 0.02 | 0.01 | 0.01 | 0.01 |
| 35 | 0.53 | 0.53 | 0.53 | 0.53 | *0.05* | 0.05 | 0.05 | 0.05 | 0.03 | 0.01 | 0.01 | 0.02 |

**S3 Table.** *δ*^13^C and *δ*^15^N in sediments from the Beidagang Wetland Nature Reserve.

| Depth(cm) | *δ*^13^C (‰) | | | | *δ*^15^N (‰) | | | |
| --- | --- | --- | --- | --- | --- | --- | --- | --- |
|  | Core 1 | Core 2 | Core 3 | Mean | Core 1 | Core 2 | Core 3 | Mean |
| 2.5 | -23.9 | -20.1 | -24.8 | -22.9 | 1.1 | 0.8 | 1.4 | 1.1 |
| 5 | -22.9 | -19.5 | -22.2 | -21.5 | 1.5 | 0.5 | 1.6 | 1.2 |
| 7.5 | -18.7 | -19.8 | -20.0 | -19.5 | 0.8 | 0.1 | 1.8 | 0.9 |
| 10 | -19.7 | -21.7 | -22.8 | -21.4 | -0.4 | 2.5 | 2.5 | 1.6 |
| 12.5 | -21.9 | -22.0 | -23.0 | -22.3 | 2.6 | 2.6 | 2.4 | 2.5 |
| 15 | -20.5 | -23.5 | -23.4 | -22.5 | 2.0 | 1.4 | 3.1 | 2.2 |
| 17.5 | -22.4 | -23.6 | -19.7 | -21.9 | 0.2 | 0.4 | 1.6 | 0.8 |
| 20 | -17.8 | -23.5 | -19.8 | -20.4 | 0.3 | -0.9 | 2.3 | 0.6 |
| 22.5 | -21.3 | -23.4 | -17.3 | -20.7 | -0.6 | 1.8 | 1.5 | 0.9 |
| 25 | -23.3 | -15.5 | -23.1 | -20.6 | 2.4 | -0.2 | 2.0 | 1.4 |
| 27.5 | -23.3 | -20.7 | -23.0 | -22.4 | 1.3 | -0.2 | 3.0 | 1.4 |
| 30 | -18.2 | -16.2 | -15.2 | -16.6 | 1.9 | 1.4 | 2.9 | 2.1 |
| 32.5 | -19.3 | -17.3 | -16.3 | -17.7 | 2.3 | -1.9 | 2.2 | 0.9 |
| 35 | -24.1 | -23.1 | -22.1 | -23.1 | 2.3 | -0.9 | 2.2 | 1.2 |
